# Retinoic acid increases the effect of bone morphogenetic protein type 2 on osteogenic differentiation of human adipose-derived stem cells

**DOI:** 10.1590/1678-7757-2018-0317

**Published:** 2019-02-21

**Authors:** Ariadne Cristiane Cabral CRUZ, Francielle Tramontini Gomes de Souza CARDOZO, Ricardo de Souza MAGINI, Cláudia Maria Oliveira SIMÕES

**Affiliations:** 1 Universidade Federal de Santa Catarina, Programa de Pós-Graduação em Odontologia, Departamento de Odontologia, Florianópolis, Santa Catarina,Brasil.; 2 Universidade de São Paulo, Departamento de Doenças Infecciosas e Parasitárias, São Paulo,Brasil.; 3 Universidade Federal de Santa Catarina, Programa de Pós-Graduação em Farmácia, Departamento de Ciências Farmacêuticas, Florianópolis, Santa Catarina,Brasil.

**Keywords:** Mesenchymal stem cells, Cell differentiation, Osteogenesis, Bone morphogenetic protein 2, Retinoic acid

## Abstract

Bone morphogenetic protein type 2 (BMP-2) and retinoic acid (RA) are osteoinductive factors that stimulate endogenous mechanisms of bone repair which can be applied on management of osseous defects in oral and maxillofacial fields.

**Objective:**

Considering the different results of RA on osteogenesis and its possible use to substitute/potency BMP-2 effects, this study evaluated the outcomes of BMP-2, RA, and BMP-2+RA treatments on i*n vitro* osteogenic differentiation of human adipose-derived stem cells (ASCs) and the signaling pathway(s) involved.

**Material and Methods:**

ASCs were treated every other day with basic osteogenic medium (OM) alone or supplemented with BMP-2, RA, or BMP-2+RA. Alkaline phosphatase (ALP) activity was determined using the r-nitrophenol method. Extracellular matrix mineralization was evaluated using von Kossa staining and calcium quantification. Expression of osteonectin and osteocalcin mRNA were determined using qPCR. Smad1, Smad4, phosphorylated Smad1/5/8, BMP-4, and BMP-7 proteins expressions were analyzed using western blotting. Signaling pathway was evaluated using the IPA^®^ software.

**Results:**

RA promoted the highest ALP activity at days 7, 14, 21, and 28, in comparison to BMP-2 and BMP-2+RA. BMP-2+RA best stimulated phosphorylated Smad1/5/8 protein expression at day 7 and Smad4 expression at days 7, 14, 21, and 28. Osteocalcin and osteonectin mRNA expressions were best stimulated by BMP-2+RA at day 7. Matrix mineralization was most improved by BMP-2+RA at days 12 and 32. Additionally, BMP-2+RA promoted the highest BMP signaling pathway activation at days 7 and 14, and demonstrated more activation of differentiation of bone-forming cells than OM alone.

**Conclusions:**

In summary, RA increased the effect of BMP-2 on osteogenic differentiation of human ASCs.

## Introduction

The repair of extensive bone defects remains a challenge in Dentistry. Given the drawbacks of the available bone graft materials, scientists have been searching for new substances and combinations to be used as osteoinductive factors. Bone morphogenetic proteins (BMPs) belong to the transforming growth factor (TGF) super-family and were initially identified by their ability to induce ectopic bone formation.^1^ BMP type 2 (BMP-2) is one of the most studied osteoinductive factors that has been used for osteogenic differentiation of mesenchymal stem cells for bone repair in oral and maxillofacial surgeries.^2^ ^,^ ^3^ However, in addition to its high cost, BMP-2 use has been related with adverse effects such as substantial swelling, erythema, and pain.^4^ ^-^ ^6^

Retinoic acid (RA) is a vitamin A-derived compound that is critical in embryonic development and maintenance of vital organs in adults.^7^ Some *in vitro* studies have shown that RA is involved in up-regulation of osteogenic genes, increasing osteogenic differentiation of different cell types including osteosarcoma-derived cells, osteoblasts, preadipocytes, and mesenchymal stem cells.^8^ ^-^ ^11^ RA has also been related to the enhancement of BMP-2 effects by increasing alkaline phosphatase (ALP) activity, RunX2 transcription factor, and osteopontin mRNA expression during chondrocytic and osteoblastic differentiation of mouse cells.^12^ ^,^ ^13^ Moreover, Cowan, et al.^14^ (2005) demonstrated that BMP-2 combined with RA accelerates *in vivo* bone formation. However, the outcomes of RA, alone or combined with BMP-2, on osteogenic differentiation are controversial, since Wang, et al.^15^ (2008) demonstrated that RA inhibits osteogenic differentiation of rat bone marrow stromal cells, Ogston, et al.^16^ (2002) and Takahashi, et al.^17^ (2008) showed a reduction on ALP activity by RA, and Hoffman, et al.^18^ (2006) verified that BMP action involves attenuation of RA signaling. Considering the different results of RA on osteogenic induction and its possible use to substitute/potency the BMP-2 effects, this study aimed to evaluate the effects of RA and BMP-2, alone or combined, on *in vitro* osteogenic differentiation of human adipose-derived stem cells (ASCs) and the signaling pathway(s) involved in the differentiation process.

## Materials and methods

### Reagents

Antibiotics/antifungicides (PSA) were purchased from Cultilab (São Paulo, SP, Brazil). All-trans retinoic acid, ascorbate, β-glycerophosphate, BMP-2, eosin, paraformaldehyde, ρ-nitrophenol phosphate (ρNPP), silver nitrate, and trypsin were obtained from Sigma-Aldrich (St. Louis, MO, USA). α-modified Eagle’s minimal essential medium (αMEM modified) and Dulbecco’s modified Eagle’s medium (DMEM) were acquired from Nutricell (São Paulo, SP, Brazil). DNAse, fetal bovine serum (FBS), SuperScript™ III, and Trizol^®^ were purchased from Invitrogen (Carlsbad, CA, USA). Calcium Assay Kit was obtained from BioAssay Systems (Hayward, CA, USA). SYBR^®^ Green PCR Master Mix was purchased from Applied Biosystems (Carlsbad, CA, USA). Immobilon-P membranes and the antibodies anti-β-actin (04-1116), -Smad1 (05-1459), -phosphorylated Smad1/5/8 (AB3848), -Smad4 (04-1033), BMP-٤ (MAB١٠٤٩), -BMP-٧ (MAB٤٣٥٠), -rabbit, and -mouse were obtained from Millipore (Danvers, MA, USA). Enhanced chemiluminescence Pierce ECL was purchased from Thermo scientific (Rockford, IL, USA). All chemicals were analytical grade.

### Adipose-derived stem cells (ASCs) isolation and cell culture conditions

Approval from the Human Ethics Committee of the Universidade Federal de Santa Catarina and written consent from all participants were obtained before commencement of this study (No. 194/06 for ASCs and No. 568/10 for osteoblasts). Human lipoaspirate tissues from healthy patients (mean age 21), with normal body mass indexes, non-smokers and not taking any medication were processed to isolate ASCs, as previously described.^19^ ASCs were maintained at sub-confluent levels and used at passage 3 for all experiments. ASCs were treated every other day, according to the groups: OM – osteogenic medium (DMEM, 10% FBS, 1% PSA, 250 µM ascorbate, and 10 mM β-glycerophosphate); BMP-2 – OM and 50 ng/ml BMP-2; RA – OM and 2.5 µM all-trans retinoic acid (RA); BMP-2+RA – OM, 50 ng/ml BMP-2, and 2.5 µM RA.^12^ Human osteoblasts isolated from biopsies of mandibular cortical bone^20^ were maintained in OM and used at passage 6 as positive control in qPCR experiments. MC3T3-E1 pre-osteoblasts subclone 4 (American Type Culture Collection, Manassas, VA, USA) were used as positive controls in ALP activity, matrix mineralization, and western blotting assays. MC3T3-E1 cells were cultured according to the supplier’s recommendations in αMEM modified with 5 µM ascorbate and 10 mM β-glycerophosphate.

Immunophenotyping and multilineage differentiation of ASCs were performed to confirm the mesenchymal stem cells characteristics, as previously described.^19^

### Determination of alkaline phosphatase activity

ASCs and MC3T3-E1 (9.4x10^4^ cells/well) were cultured in 24-well plates for 7, 14, 21, and 28 days. ALP activity was determined by releasing ρ-nitrophenol (ρNP) from ρ-nitrophenol phosphate (ρNPP) as previously described.^20^ ALP activity was calculated from a standard ρNP curve and all values were normalized against total protein concentration determined by Lowry’s method.^21^

### Evaluation of matrix mineralization

Von Kossa staining was applied to detect the presence of phosphate, an indicator of extracellular matrix mineralization. ASCs and MC3T3-E1 (2x10^4^ cells/well) were cultured in 96-well plates for 21 days. Cells were fixed for 1h with 3% (v/v) aqueous paraformaldehyde, stained with 1% (w/v) silver nitrate under light exposure for 1h, and counterstained with eosin.^20^

Total calcium was quantified on ASCs and MC3T3-E1 (2x10^4^ cells/well), cultured in 96-well plates for 5, 12, 23, and 32 days, using a Calcium Assay Kit.^20^

### qPCR

ASCs and human osteoblasts (9.4x10^4^ cells/well) were cultured in 24-well plates for 7, 14, 21, and 28 days. Total RNA was isolated using Trizol^®^ reagent, according to the manufacturer’s instructions. RNA concentration was estimated by NanoVue (GE Healthcare, Little Chalfont, Buckingmshire, UK). Only samples with an optical density ratio at 260 nm/280 nm greater than 1.8 were used. After DNAse treatment, total RNA (1 µg) was transcribed into cDNA using oligo dT primers and SuperScript, according to the manufacturer’s instructions. PCR amplifications were performed in a 20 µl reaction mix containing 10 ng cDNA, 50 nM primers, and 10 µl Power SYBR1 Green PCR Master Mix. Comparative cycle threshold (Ct) method was used to quantify changes in osteocalcin and osteonectin gene expression between control (human osteoblasts) and treated ASCs. Fold change in transcript levels was calculated according to the 2-∆∆Ct method, where ∆∆Ct = (∆Ct control) - (∆Ct treatment) and ∆Ct = (Ct target gene) - (Ct internal control; ribosomal 18s). The following primers were used: Osteocalcin mRNA relative expression: 5’-AGGGCAGCGAGGTAGTGAAG-3’ (forward); 5’-AACTCGTCACAGTCCGGATTG-3’ (reverse). Osteonectin mRNA expression: 5’-CGGGTGAAGAAGATCCATGAG-3’ (forward); 5’-CTGCCAGTGTACAGGGAAGATG-3’ (reverse).^20^

### Western blotting

ASCs and MC3T3-E1 (9.4x10^4^ cells/well) were cultured in 24-well plates for 7, 14, 21, and 28 days. Samples (5 µg of protein) were separated electrophoretically on SDS/polyacrylamide gel and transferred to Immobilon-P membranes (Millipore, Danvers, MA, USA). After blocking, membranes were incubated overnight using anti-Smad1 (05-1459, 1:500), or -Smad4 (04-1033, 1:1000), or -phosphorylated Smad1/5/8 (AB3848, 1:500), or -BMP-4 (MAB1045, 1:500), or -BMP-7 (MAB4350, 2 µg /ml), or -β actin (04-1116, 1:2000) antibodies. After incubation with each corresponding secondary antibody, the Enhanced chemiluminescence *Pierce ECL* substrate was used for detection, according to the manufacturer’s protocol. Expression of each protein was normalized against β-actin. Phosphorylated Smad1/5/8 was normalized against Smad1. Band intensities were quantified using the ImageJ software (National Institute of Health, USA).^20^

### Signaling pathways analysis

Calcium, proteins (ALP, Smad1, Smad4, phosphorylated Smad1/5/8, BMP4, and BMP7) and mRNAs (osteonectin and osteocalcin), designed as analytes, were analyzed using Ingenuity Pathways Analysis (IPA^®^, QIAGEN Redwood City, USA, www.ingenuity.com) to determine cellular pathways and biological functions impacted by BMP-2, RA, or BMP2+RA supplementation at all evaluated time points. All analytes with their corresponding quantification values, gene ID, and ρ values were uploaded into the software to run IPA-core analysis. Overall dysregulated analytes (up-regulated and down-regulated; both together) were analyzed. Groups were compared to each other (designed as observation) by fold change of analyte levels. Results identified the canonical pathways and biological functions that were most significant to the data set. Right-tailed Fisher’s exact test was used to calculate a ρ value that determined the probability of each pathway or biological function assigned to that data set being caused by chance alone. The prediction of activation or inhibition of a canonical pathway or biological function was based on z-score. IPA^®^ automatically calculates z-scores based on differentially expressed analytes in our dataset and the information stored in Ingenuity’s Knowledge Base.^22^ ^,^ ^23^

### Statistical analysis

Three independent experiments were performed to confirm the reproducibility of the results. Graph Pad Software Inc. (San Diego, CA, USA) was used for statistical evaluation. ALP activity, calcium quantification, mRNA relative expression, and protein quantification results from all groups were compared through one-way analysis of variance (ANOVA) followed by Tukey’s *post hoc* test. Differences between datasets with ρ<0.05 were considered statistically significant. Statistical analyses were performed comparing all treatments at each time point separately.

## Results

The confirmation that the ASCs used in this report were mesenchymal stem cells was previously published.^19^

### RA increased ALP activity

**Figure 1:**
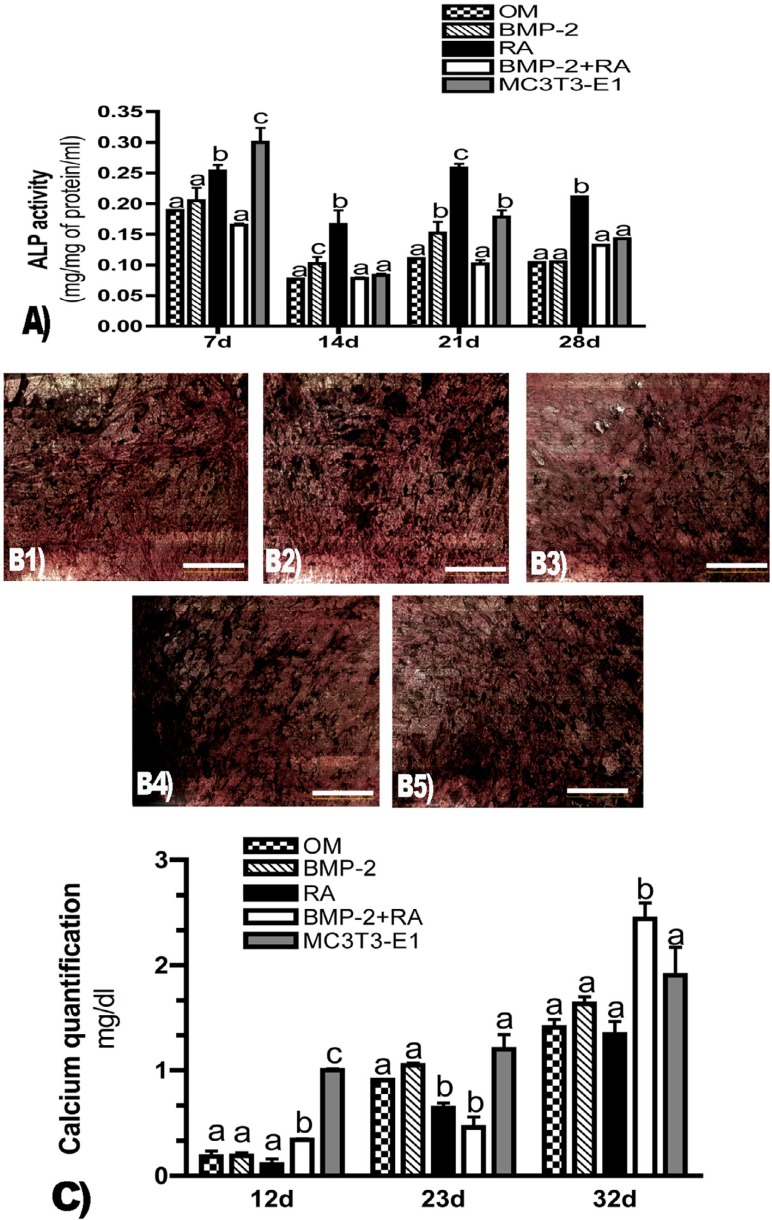
Extracellular matrix evaluation. A) Alkaline phosphatase (ALP) activity expressed as mg of rNitrophenol (rNP) per mg of protein per ml at days 7, 14, 21, and 28. Different letters refer to statistically significant differences (r<0.001) (ANOVA/Tukey). Statistical analyses were performed comparing all treatments at each time point separately. B) von Kossa staining of ASCs cultured for 21 days. Bar = 500 µm. Black areas show the presence of phosphate and indicate extracellular matrix mineralization, suggesting differentiation into osteoblasts. Cells were treated with OM (B1), OM supplemented with BMP-2 (B2), OM with RA (B3), or OM supplemented with BMP-2 and RA (B4). MC3T3-E1 was used as a positive control (B5). Images are representative of three independent experiments; C) Calcium quantification expressed as mg per dl at days 12, 23, and 32. Bars represent the mean ± SD of three independent experiments. Different letters represent statistically significant differences (r<0.05) (ANOVA/Tukey) among treatments at each time point.

ALP activity peaked for all groups at day 7 of culturing, when MC3T3-E1 showed the highest quantification level. RA promoted the highest ALP activity at all time points (days 7, 14, 21, and 28) (ρ<0.001) when compared to BMP-2 and BMP-2+RA treatments (Figure 1a).

### BMP-2+RA increased calcium deposition at extracellular matrix

Von Kossa staining revealed all groups displayed detectable extracellular matrix mineralization at day 21 (Figure 1b1-b5).

Calcium quantification showed no detectable deposition at extracellular matrix at day 5 (data not shown). At day 12, control MC3T3-E1 showed the highest calcium production; among the tested groups, BMP-2+RA treatment promoted significantly the highest calcium levels (ρ<0.0001). At day 23, OM, BMP-2, and MC3T3-E1 demonstrated similar calcium levels, which increased significantly when compared to RA and BMP-2+RA groups (ρ<0.0001). At day 32, BMP-2+RA group exhibited the highest calcium levels when compared to the test and control groups (ρ<0.0005) (Figure 1c).

### BMP-2+RA increased osteonectin and osteocalcin mRNA expression

**Figure 2:**
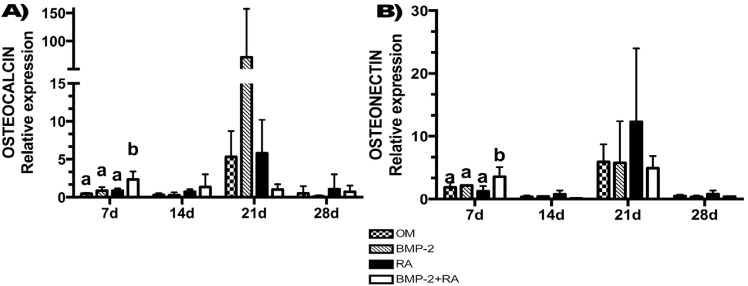
mRNA relative expressions. ASCs were treated according to the groups: OM (osteogenic medium); BMP-2 (OM and BMP-2); RA (OM and retinoic acid, RA); BMP-2+RA (OM, BMP-2, and RA). Results were normalized against human osteoblasts (positive control). Different letters represent statistically significant differences among treatments at each time point (ANOVA/Tukey, ρ<0.05). Bars represent the mean ± SD of three independent experiments

At day 7, BMP-2+RA showed the highest osteocalcin (ρ=0.0042) and osteonectin (ρ=0.0030) mRNA expression levels. There was no difference on osteocalcin and osteonectin mRNA expression among all groups at days 14 (ρ=0.3317; ρ=0.0983, respectively), 21 (ρ=0.1154; ρ=0.4422, respectively), and 28 (ρ=0.7041; ρ=0.2414, respectively) (Figure 2a and Figure 2b).

### Expression of osteoblast-related proteins

**Figure 3:**
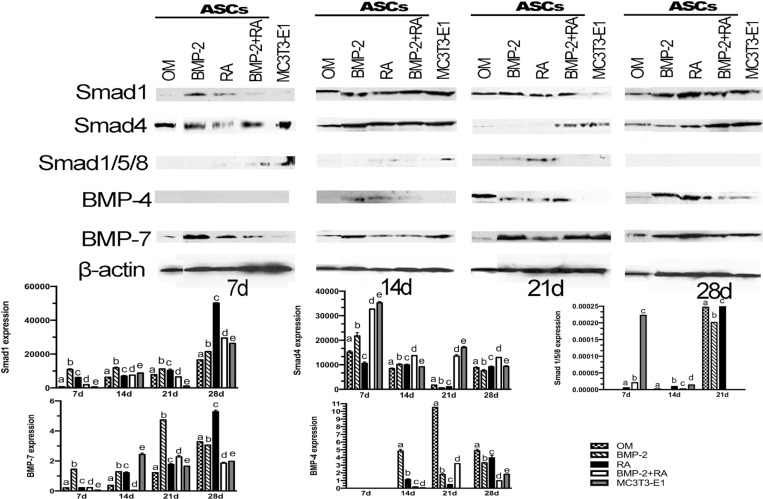
Expression of osteoblast-related proteins. Proteins expressions evaluated by western blotting at days 7, 14, 21, and 28 of ASCs treated according to the groups: OM (osteogenic medium); BMP-2 (OM and BMP-2); RA (OM and retinoic acid, RA); BMP-2+RA (OM, BMP-2, and RA). MC3T3-E1 was used as positive control. Bands are representative of three independent experiments. Band intensities were quantified. Different letters represent statistically significant differences (r<0.05) (ANOVA/Tukey) among treatments at each time point

According to Figure 3, at day 7, BMP-2 treated group expressed the highest Smad1 level, followed by RA, BMP-2+RA, OM, and MC3T3-E1 groups (ρ<0.0001). At day 14, the highest expression was demonstrated by BMP-2, followed by MC3T3-E1, BMP-2+RA, RA, and OM (ρ<0.0001). At day 21, BMP-2 showed the highest Smad1 expression, followed by RA, OM, BMP-2+RA, and MC3T3-E1 (ρ<0.0001). Finally, RA demonstrated the highest expression at day 28, followed by BMP-2+RA, MC3T3-E1, BMP-2, and OM (ρ<0.0001).

Regarding Smad4, control cells MC3T3-E1 demonstrated the highest expression levels, followed by ASCs treated with BMP-2+RA, BMP-2, OM, and RA (ρ<0.0001) at day 7. At day 14, BMP-2+RA treatment showed the highest expression levels, followed by BMP-2, RA, MC3T3-E1, and OM (ρ<0.0001). At day 21, MC3T3-E1 exhibited the highest expression level followed by BMP-2+RA, OM, RA, and BMP-2 treatments (ρ<0.0001). At day 28, BMP-2+RA group showed the highest Smad4 expression, followed by MC3T3-E1, RA, OM, and BMP-2 groups (ρ<0.0001).

MC3T3-E1 exhibited the highest expression of phosphorylated Smad1/5/8 at days 7 (ρ<0.001). Among the test groups, BMP-2+RA expressed more of these proteins at day 7, followed by RA (ρ<0.001). At day 14, RA treatment induced the highest expression of phosphorylated Smad1/5/8, followed by BMP-2+RA (ρ<0.001). There was no expression of these proteins in the OM group at day 7, and BMP-2 group at days 7 and 14. At day 21, RA treatment exhibited the highest phosphorylated Smad1/5/8 level, followed by OM and BMP-2 (ρ<0.001), while no expression was observed in the BMP-2+RA and MC3T3-E1 groups. These phosphorylated Smads were not detected in any group at day 28.

Considering BMP-4, no group was able to express this growth factor at day 7. At day 14, BMP-2 demonstrated the highest BMP-4 level, followed by RA, BMP-2+RA, MC3T3-E1, and OM (ρ<0.0001). At day 21, the OM group exhibited the highest BMP-4 expression, followed by BMP-2+RA, BMP-2, and RA groups (ρ<0.0001). OM treatment demonstrated the highest BMP-4 level, followed by RA, BMP-2, MC3T3-E1, and BMP-2+RA at day 28 (ρ<٠.٠٠٠١).

At day 7, the BMP-2 treated group expressed the highest BMP-7 amount, followed by BMP-2+RA, RA, OM, and MC3T3-E1 groups (ρ<0.0001). The highest BMP-7 expression was exhibited by MC3T3-E1, followed by BMP-2, RA, OM, and BMP-2+RA at day 14 (ρ<0.0001). At day 21, BMP-2 demonstrated the highest BMP-7 expression, followed by BMP-2+RA, RA, MC3T3-E1, and OM groups (ρ<0.0001). Finally, RA showed the highest expression at day 28, followed by OM, BMP-2, MC3T3-E1, and BMP-2+RA (ρ<0.0001).

### Signaling pathways analysis

IPA^®^ analysis indicated that the major changes in BMP signaling pathway occurred at days 14 and 21 (Table 1). Osteogenic differentiation peaked at day 14 for the positive control group (MC3T3-E1). When compared to the negative control (OM), BMP-2+RA, BMP-2, and RA treatments promoted the most BMP signaling pathway up-regulation at day 14. At days 7, 14, and 21, BMP-2 treatment induced BMP signaling pathway more strongly than the RA treatment. Overall, among the tested groups, the BMP-2+RA combination more actively induced BMP signaling in most of the set points (days 7, 14, and 28).

**Table 1:** Relation between the different cell treatments and the BMP signaling pathway indicated by IPA® software at days 7, 14, 21, and 28. ASCs were treated according to the groups: OM (osteogenic medium); BMP-2 (OM and BMP-2); RA (OM and retinoic acid, RA); BMP-2+RA (OM, BMP-2, and RA). MC3T3-E1 was used as positive control. r<0.05 represents statistically significant difference. Positive z-score indicates up-regulation (highlighted in orange) and negative z-score implies down-regulation (highlighted in blue) of the signaling pathway evidenced by the IPA® software (v=versus)

**BMP signaling pathway – Day 7**			**BMP signaling pathway – Day 21**		
**Observation**	**Z-score**	**ρ value**	**Observation**	**Z-score**	**ρ value**

OM v MC3T3-E1	0		OM v MC3T3-E1	0	
BMP-2 v MC3T3-E1	0		BMP-2 v MC3T3-E1	0	
RA v MC3T3-E1	0		RA v MC3T3-E1	0	
BMP-2+RA v MC3T3-E1	0		BMP-2+RA v MC3T3-E1	0	
BMP-2 v OM	0		BMP-2 v OM	1.342	5.14E-11
RA v OM	0		RA v OM	0.447	5.14E-11
BMP-2+RA v OM	0		BMP-2+RA v OM	-1.342	5.14E-11
BMP-2 v RA	1	4.51E-09	BMP-2 v RA	0.447	5.14E-11
BMP-2 v BMP-2+RA	-1	4.51E-09	BMP-2 v BMP-2+RA	1.342	5.14E-11
RA v BMP-2+RA	-1	4.51E-09	RA v BMP-2+RA	0.447	5.14E-11

**BMP signaling pathway – Day 14**			**BMP signaling pathway – Day 28**		
**Observation**	**Z-score**	**ρ value**	**Observation**	**Z-score**	**ρ value**

OM v MC3T3-E1	-2	4.41E-9	OM v MC3T3-E1	0	
BMP-2 v MC3T3-E1	0		BMP-2 v MC3T3-E1	0	
RA v MC3T3-E1	-1	4.41E-9	RA v MC3T3-E1	0	
BMP-2+RA v MC3T3-E1	-1	8.8E-9	BMP-2+RA v MC3T3-E1	0	
BMP-2 v OM	1	5.14E-11	BMP-2 v OM	0	
RA v OM	2.236	5.14E-11	RA v OM	1	8.8E-9
BMP-2+RA v OM	1.342	4.41E-9	BMP-2+RA v OM	2	8.8E-9
BMP-2 v RA	1	8.8E-9	BMP-2 v RA	-1	8.8E-9
BMP-2 v BMP-2+RA	-1	8.8E-9	BMP-2 v BMP-2+RA	-2	8.8E-9
RA v BMP-2+RA	-0.447	5.14E-11	RA v BMP-2+RA	0	

**DIFFERENTIATION OF BONE-FORMING CELLS at day 14**					
**Observation**	**ρ value**	**Activation z-score**	**Molecules**		

OM v MC3T3-E1	2.20E-06	-2	Ca2+,SMAD1,SMAD4,SMAD5		
RA v OM	1.24E-07	2	Ca2+,SMAD1,SMAD4,SMAD5		
BMP-2+RA v OM	1.24E-07	2	Ca2+,SMAD1,SMAD4,SMAD5		

In addition to the identification of signaling pathway involved in the osteogenic differentiation, IPA^®^ analysis suggested the biological functions influenced by the treatments (Table 1). According to these results, MC3T3-E1, RA, and BMP-2+RA increased the differentiation of bone-forming cells when compared to OM at day 14.

## Discussion

Despite many efforts, bone repair remains a challenge in many cases of oral and maxillofacial surgeries. BMP-2 has been clinically applied to stimulate osteogenic differentiation of mesenchymal stem cells and to promote bone repair. In addition to its high cost, BMP-2 use has been related with adverse effects.^4^ ^-^ ^6^ Given that and the possible use of RA to substitute and/or potency BMP-2 effects, this study compared the outcomes of BMP-2, RA, and BMP-2+RA on *in vitro* osteogenic differentiation of ASCs and evaluated the BMP signaling pathways involved in the differentiation process. Among the groups, RA most stimulated the ALP activity at days 7, 14, 21, and 28. BMP-2+RA most stimulated the matrix mineralization at days 12 and 32; osteocalcin and osteonectin mRNA expression at day 7; Smad4 expression at days 7, 14, 21, and 28; and phosphorylated Smad1/5/8 expression at day 7. Additionally, BMP-2+RA promoted the highest BMP signaling pathway activation at days 7 and 14. Furthermore, at day 14, RA and BMP-2+RA treatments increased the activation of differentiation of bone-forming cells when compared to OM, while BMP-2 alone did not demonstrate this capacity.

These results increase the possibility of using RA combined with BMP-2 to enhance the osteogenic differentiation. Corroborating our results, Wan, et al.^12^ (2006) observed the increase in osteogenic differentiation of ASCs with the association of BMP-2 and RA. Li, et al.^13^ (2003) observed that RA stimulates chondrocyte differentiation and enhances BMP effects. Cowan, et al.^14^ (2005) demonstrated that BMP-2 combined with RA accelerates *in vivo* bone formation. Interestingly, Bi, et al.^24^ (2018) suggest that the combination of RA and BMP2/7 could be a novel approach to treat hyperactive osteoclast-induced bone loss such as in inflammation-induced severe osteoporosis and bone loss caused by cancer metastasis to bone. Conversely, Hoffman, et al.^18^ (2006) verified the attenuation of RA signaling from BMP action.

RA exhibited higher ALP activity than BMP-2 and BMP-2+RA at all time points, and than positive control at most time points. Some studies^8^ ^,^ ^25^ also showed that RA stimulates ALP activity. Conversely, other studies^16^ ^,^ ^17^ ^,^ ^26^ did not observe such stimulation on ALP activity by RA. Some reports describe the potentiation between RA and BMP-2 on ALP activity^8^ ^,^ ^12^ ^,^ ^27^. Such potentiation was not observed in this study.

We performed the von Kossa staining to evaluate osteogenic differentiation. In this technique, the stain reacts with phosphate in the presence of acidic material and does not react with calcium. Despite this method being used in several reports, this is not a sensitive assay to evaluate mineralization. For this reason, we also quantified the calcium at the extracellular matrix^28^. Corroborating other studies^9^ ^,^ ^12^, BMP-2 and RA potency each other’s effects on calcium secretion. Additionally, at the mineral deposition peak (day 32), calcium concentration in BMP-2+RA group was higher than in the positive control. This cooperation might occur due to the inhibition of adipocyte differentiation, as previously stated^8^. In contrast, Bi, et al.^27^ (2013) observed additional matrix mineralization promoted by BMP when compared to BMP combinedwithRA.

Osteocalcin and osteonectin are important noncollagen bone matrix components considered markers of osteoblast mineralization^29^. Our results showed that BMP-2+RA stimulated osteonectin and osteocalcin mRNA expression at day 7. Such early osteocalcin and osteonectin mRNA expression could be connected to the highest mineralization at extracellular matrix promoted by the association of BMP-2 and RA, even with the low ALP expression observed in this group. When studying preadipocytes, Skillington, et al.^8^ (2002) observed that BMP-2, alone or combined with RA, was unable to induce neither osteocalcin mRNA expression nor extracellular matrix mineralization. Wang, et al.^15^ (2008) verified that bone marrow stromal cells treated with RA did not enlarge osteonectin mRNA expression. On the other hand, other reports^25^ ^,^ ^30^ found that RA stimulated the osteocalcin mRNA expression. Bi, et al.^27^ (2013) showed fewer osteocalcin expression in cells treated with RA combined with BMP-2/7 when compared to cells treated with BMP-2/7 only. These different findings could be due to the different cell types analyzed. Our report is the first to evaluate the effects of BMP+RA on osteogenic differentiation of human ASCs.

In addition to evaluating osteogenic differentiation of ASCs, we studied the effect of different treatments on Smads expression. Classical signaling pathways for BMPs begins with the binding of BMP to a dimeric complex of transmembrane serine–threonine kinase receptors, type I and type II receptors. Type I receptors include BMP receptors (BMPRs) -Ia, -Ib, and activin receptor type 1 (ACTR1); type II receptors include BMPR-II, ACTR2, and -2b. Heteromeric receptor complexes comprising type I and II receptors lead to ligand-induced phosphorylation of type I receptors. Following the activation of this receptor, receptor kinases phosphorylate transcription factors Smad1, 5, or 8 that subsequently form heteromeric complexes with Smad4 and activate the expression of target genes in the nucleus^31^ ^,^ ^32^. Herein, ASCs treated with BMP-2+RA showed more Smad4 expression than cells treated with BMP-2 or RA alone. Conversely, ASCs treated with BMP-2 exhibited more Smad1 expression than RA alone or combined with BMP-2. However, the analysis of phosphorylated Smad1/5/8 is much more important than Smad1 since phosphorylation indicates the activation of these proteins. We showed that BMP-2+RA up-regulated phosphorylated Smad1/5/8 expressions at the beginning of the differentiation process. Moreover, ASCs treated with BMP-2+RA demonstrated a similar expression pattern to positive control, discontinuing the expression of phosphorylated Smads from day 21 onwards, which may be related to an osteoblast lineage commitment. The IPA^®^ analysis corroborates the molecular mechanism behind the cross talk of RA and BMP is the BMP signaling pathway.

The interpretation of osteogenic differentiation based on analysis of osteogenic markers separately is very challenging due to the difficulty of understanding and correlating all results with the whole differentiation process. In this study, we applied the IPA^®^ software to evaluate the BMP signaling pathways involved in osteogenic differentiation and to observe the process more comprehensively. IPA^®^ calculates the probability that the analytes are involved in particular pathways, comparing it to the total number of occurrences of those analytes in all function annotation stored in the Ingenuity Knowledge Base. We confirmed that both RA and BMP-2, alone or combined, used the BMP signaling pathway for osteogenic differentiation^33^. Adams, et al.^34^ (2003) showed that RA seemed to stimulate type-X collagen gene transcription, partially by stimulating the BMP signaling pathway and partially through independent mechanisms. Liu, et al.^11^ (2014) observed RA modulated osteogenesis through the Wnt/β-catenin signaling pathway. Conversely, Sheng, et al.^35^ (2010) proposed that RA enhances the interaction between phosphorylated Smad1 and its ubiquitin E3 ligases, resulting in phosphorylated Smad1 ubiquitination and proteasomal degradation, suggesting a mechanism through which RA suppresses BMP/Smad signaling. Zhang, et al.^36^ (2017) demonstrated that RA signaling regulates COL9A1 expression through the BMP2-WNT4-RUNX1 pathway. These different results may be occur because the BMP signaling pathway is cell type-specific and cell context-dependent, given that Yang, et al.^33^ (2012) studied 143B osteosarcoma cells, Adams, et al.^34^ (2003) evaluated chondrocytes from embryonic chick sterna, Liu, et al.^11^ (2014) employed 3-T3-L1 preadipocytes, Sheng, et al.^35^ (2010) evaluated P19C6 cells, a subclone of mouse embryonic carcinoma, and Zhang, et al.^36^ (2017) studied antler chondrocytes.

We also observed that BMP-2+RA up-regulated the BMP signaling pathway when compared to BMP-2 and RA treatments at days 7 and 14. Conversely, at day 21, BMP-2 and RA up-regulated the BMP signaling pathway when compared to BMP-2+RA. The reason for this finding may be related to an early osteoblast lineage commitment of ASCs under BMP-2+RA treatment, discontinuing the osteogenic markers expression, similar to what we suggested in the results for phosphorylated Smads. According to IPA^®^ results, RA and BMP-2+RA treatments increased the activation of differentiation of bone-forming cells when compared to the negative control (OM), while BMP-2 did not demonstrate this capacity.

## Conclusions

Results indicate that BMP-2+RA better induced osteogenic differentiation of human ASCs. This finding strengthens the possibility of using BMP-2 combined with RA to enhance bone repair in oral and maxillofacial surgery. Additionally, this combination may enable the use of reduced doses of BMP-2, possibly minimizing BMP-2-induced adverse effects and treatment costs. Future experiments evaluating the capacity of BMP-2 combined with RA to enhance bone repair in animals must be conducted, and further research is required to determine the synergism mechanisms between BMP-2 and RA in bone repair.

## References

[1] Urist MR (1965). Bone: formation by autoinduction. Science.

[2] Alonso N, Tanikawa DY, Freitas RS, Canan L, Ozawa TO, Rocha DL (2010). Evaluation of maxillary alveolar reconstruction using a resorbable collagen sponge with recombinant human bone morphogenetic protein-2 in cleft lip and palate patients. Tissue Eng Part C Methods.

[3] Wang J, Guo J, Liu J, Wei L, Wu G (2014). BMP-functionalised coatings to promote osteogenesis for orthopaedic implants. Int J Mol Sci.

[4] Shah MM, Smyth MD, Woo AS (2008). Adverse facial edema associated with off-label use of recombinant human bone morphogenetic protein-2 in cranial reconstruction for craniosynostosis. J Neurosurg Pediatr.

[5] Boyne PJ, Marx RE, Nevins M, Triplett G, Lazaro E, Lilly LC (1997). A feasibility study evaluating rhBMP-2/absorbable collagen sponge for maxillary sinus floor augmentation. Int J Periodontics Restorative Dent.

[6] Vaidya R, Carp J, Sethi A, Bartol S, Craig J, Les CM (2007). Complications of anterior cervical discectomy and fusion using recombinant human bone morphogenetic protein-2. Eur Spine J.

[7] Conaway HH, Henning P, Lerner UH (2013). Vitamin A metabolism, action, and role in skeletal homeostasis. Endocr Rev.

[8] Skillington J, Choy L, Derynck R (2002). Bone morphogenetic protein and retinoic acid signaling cooperate to induce osteoblast differentiation of preadipocytes. J Cell Biol.

[9] Zhang W, Deng ZL, Chen L, Zuo GW, Luo Q, Shi Q (2010). Retinoic acids potentiate BMP9-induced osteogenic differentiation of mesenchymal progenitor cells. PLoS One.

[10] Zhang L, Zhou Q, Zhang N, Li W, Ying M, Ding W (2014). E2F1 impairs all-trans retinoic acid-induced osteogenic differentiation of osteosarcoma via promoting ubiquitination-mediated degradation of RARα. Cell Cycle.

[11] Liu Y, Liu Y, Zhang R, Wang X, Huang F, Yan Z (2014). All-trans retinoic acid modulates bone morphogenic protein 9-induced osteogenesis and adipogenesis of preadipocytes through BMP/Smad and Wnt/β-catenin signaling pathways. Int J Biochem Cell Biol.

[12] Wan DC, Shi YY, Nacamuli RP, Quarto N, Lyons KM, Longaker MT (2006). Osteogenic differentiation of mouse adipose-derived adult stromal cells requires retinoic acid and bone morphogenetic protein receptor type IB signaling. Proc Natl Acad Sci U S A.

[13] Li X, Schwarz EM, Zuscik MJ, Rosier RN, Ionescu AM, Puzas JE (2003). Retinoic acid stimulates chondrocyte differentiation and enhances bone morphogenetic protein effects through induction of Smad1 and Smad5. Endocrinology.

[14] Cowan CM, Aalami OO, Shi Y-Y, Chou Y-F, Mari C, Thomas R (2005). Bone morphogenetic protein 2 and retinoic acid accelerate in vivo bone formation, osteoclast recruitment, and bone turnover. Tissue Eng.

[15] Wang A, Ding X, Sheng S, Yao Z (2008). Retinoic acid inhibits osteogenic differentiation of rat bone marrow stromal cells. Biochem Biophys Res Commun.

[16] Ogston N, Harrison A, Cheung HF, Ashton B, Hampson G (2002). Dexamethasone and retinoic acid differentially regulate growth and differentiation in an immortalised human clonal bone marrow stromal cell line with osteoblastic characteristics. Steroids.

[17] Takahashi T, Kamiya N, Kawabata N, Takagi M (2008). The effect of retinoic acid on a zinc finger transcription factor, AJ18, during differentiation of a rat clonal preosteoblastic cell line, ROB-C20, into osteoblasts. Arch Oral Biol.

[18] Hoffman LM, Garcha K, Karamboulas K, Cowan MF, Drysdale LM, Horton WA (2006). BMP action in skeletogenesis involves attenuation of retinoid signaling. J Cell Biol.

[19] Cruz AC, Caon T, Menin A, Granato R, Boabaid F, Simões CMO (2015). Adipose-derived stem cells incorporated into platelet-rich plasma improved bone regeneration and maturation in vivo. Dent Traumatol.

[20] Cruz AC, Silva ML, Caon T, Simões CM (2012). Addition of bone morphogenetic protein type 2 to ascorbate and beta-glycerophosphate supplementation did not enhance osteogenic differentiation of human adipose-derived stem cells. J Appl Oral Sci.

[21] Lowry OH, Rosebrough NJ, Farr AL, Randall RJ (1951). Protein measurement with the Folin phenol reagent. J Biol Chem.

[22] Rai R, Chauhan SK, Singh VV, Rai M, Rai G (2016). RNA-seq analysis reveals unique transcriptome signatures in systemic lupus erythematosus patients with distinct autoantibody specificities. PLoS One.

[23] Mullapudi N, Ye B, Suzuki M, Fazzari M, Han W, Shi MK (2015). Genome wide methylome alterations in lung cancer. PLoS One.

[24] Bi W, Liu Y, Guo J, Lin Z, Liu J, Zhou M (2018). All-trans retinoic-acid inhibits heterodimeric bone morphogenetic protein 2/7-stimulated osteoclastogenesis, and resorption activity. Cell Biosci.

[25] Yu Y, Al-Mansoori L, Opas M (2015). Optimized osteogenic differentiation protocol from R1 mouse embryonic stem cells in vitro. Differentiation.

[26] Bosetti M, Sabbatini M, Calarco A, Borrone A, Peluso G, Cannas M (2016). Effect of retinoic acid and vitamin D3 on osteoblast differentiation and activity in aging. J Bone Miner Metab.

[27] Bi W, Gu Z, Zheng Y, Wang L, Guo J, Wu G (2013). Antagonistic and synergistic effects of bone morphogenetic protein 2/7 and all-trans retinoic acid on the osteogenic differentiation of rat bone marrow stromal cells. Dev Growth Differ.

[28] Bonewald LF, Harris SE, Rosser J, Dallas MR, Dallas SL, Camacho NP (2003). Von Kossa staining alone is not sufficient to confirm that mineralization in vitro represents bone formation. Calcif Tissue Int.

[29] Ivanovski S, Haase HR, Bartold PM (2001). Expression of bone matrix protein mRNAs by primary and cloned cultures of the regenerative phenotype of human periodontal fibroblasts. J Dent Res.

[30] Yan Q, Li Y, Cheng N, Sun W, Shi B (2015). Effect of retinoic acid on the function of lipopolysaccharide-stimulated bone marrow stromal cells grown on titanium surfaces. Inflamm Res.

[31] Fujii M, Takeda K, Imamura T, Aoki H, Sampath TK, Enomoto S (1999). Roles of bone morphogenetic protein type I receptors and Smad proteins in osteoblast and chondroblast differentiation. Mol Biol Cell.

[32] Song B, Estrada KD, Lions KM (2009). Smad signaling in skeletal development and regeneration.

[33] Yang QJ, Zhou LY, Mu YQ, Zhou QX, Luo JY, Cheng L (2012). All-trans retinoic acid inhibits tumor growth of human osteosarcoma by activating Smad signaling-induced osteogenic differentiation. Int J Oncol.

[34] Adams SL, Pallante KM, Niu Z, Cohen AJ, Lu J, LeBoy PS (2003). Stimulation of type-X collagen gene transcription by retinoids occurs in part through the BMP signaling pathway. J Bone Joint Surg Am.

[35] Sheng N, Xie Z, Wang C, Bai G, Zhang K, Zhu Q (2010). Retinoic acid regulates bone morphogenic protein signal duration by promoting the degradation of phosphorylated Smad1. Proc Natl Acad Sci.

[36] Zhang HL, Guo B, Yang ZQ, Duan CC, Geng S, Wang K (2017). ATRA signaling regulates the expression of COL9A1 through BMP2-WNT4-RUNX1 pathway in antler chondrocytes. J Exp Zool Part B Mol Dev Evol.

